# Neural Representations of Personally Familiar and Unfamiliar Faces in the Anterior Inferior Temporal Cortex of Monkeys

**DOI:** 10.1371/journal.pone.0018913

**Published:** 2011-04-15

**Authors:** Satoshi Eifuku, Wania C. De Souza, Ryuzaburo Nakata, Taketoshi Ono, Ryoi Tamura

**Affiliations:** 1 Department of Integrative Neuroscience, Graduate School of Medicine and Pharmaceutical Sciences, University of Toyama, Toyama, Japan; 2 Department of Judo Neurophysiotherapy, Graduate School of Medicine and Pharmaceutical Sciences, University of Toyama, Toyama, Japan; The University of Hong Kong, Hong Kong

## Abstract

To investigate the neural representations of faces in primates, particularly in relation to their personal familiarity or unfamiliarity, neuronal activities were chronically recorded from the ventral portion of the anterior inferior temporal cortex (AITv) of macaque monkeys during the performance of a facial identification task using either personally familiar or unfamiliar faces as stimuli. By calculating the correlation coefficients between neuronal responses to the faces for all possible pairs of faces given in the task and then using the coefficients as neuronal population-based similarity measures between the faces in pairs, we analyzed the similarity/dissimilarity relationship between the faces, which were potentially represented by the activities of a population of the face-responsive neurons recorded in the area AITv. The results showed that, for personally familiar faces, different identities were represented by different patterns of activities of the population of AITv neurons irrespective of the view (e.g., front, 90° left, etc.), while different views were not represented independently of their facial identities, which was consistent with our previous report. In the case of personally unfamiliar faces, the faces possessing different identities but presented in the same frontal view were represented as similar, which contrasts with the results for personally familiar faces. These results, taken together, outline the neuronal representations of personally familiar and unfamiliar faces in the AITv neuronal population.

## Introduction

Various types of information are embedded in faces, and this information is critically important for daily non-verbal communication between primate con-specifics [1]. It has been suggested that a neural circuitry specialized for the processing of faces exists in the primate brain, by non-human primate single-cell recording studies which have shown the existence of face-responsive neurons [2]–[8] and by human functional brain imaging studies [9]–[11], which have shown the existence of face-responsive areas. Recently, it was demonstrated that faces are represented in some discrete patch-like organizations in the temporal cortex of macaque monkeys [12]–[15]. In addition, we have already reported that the ventral portion of the anterior inferior temporal cortex (AITv) in monkeys showed selectivity to identities of faces and suggested that the area is crucial for face identification [16], [17].

Among the various types of facial information embedded in faces, it has been shown in a number of previous studies that the personal familiarity of a face is critically important in face processing [18]. More specifically, behavioral measures, such as reaction time, are usually significantly faster for personally familiar than unfamiliar faces [19]. The personal familiarity of faces viewed is defined by whether or not the subjects have encountered the depicted individual in the real world. Several non-invasive studies in humans have been conducted so far to elucidate the neural basis for the discrimination of personal familiarity or unfamiliarity [20]–[27]. However, no single-cell recording studies in monkeys have been performed. A single-cell recording study in monkeys provides a strong basis for characterizing the neural representations composed by individual neurons; our aim was to conduct this characterization based on single-cell recordings for personal familiarity or unfamiliarity.

Personal familiarity is considered to be acquired through learning after birth, by repeated experiences with other individuals such as caretakers. It should be noted in this context that a number of previous studies have demonstrated significant effects of visual learning (or experience) on neural representations of visual items in the anterior inferior temporal cortex (AIT) [28]–[36]. Based on these previous findings, we hypothesized that a substantial difference should exist between the neural representations of personally familiar and unfamiliar faces.

In the present study we analyzed representations of personally familiar or unfamiliar faces by the population of face-responsive neurons in the AITv. The results in the present study not only extend our previous finding that facial identities irrespective of facial view are represented by the AITv area, but also newly characterize neural representations of personally unfamiliar faces.

## Methods

### Subjects and Ethics

Two female Japanese macaque monkeys (*Macaca fuscata*, 4–7 kg body weight), which were designated as monkey A and monkey B, were used for the experiment. All experimental protocols were approved by the Animal Care and Use Committee, University of Toyama (Permit # MED-46), and all animal protocols conformed with the National Institutes of Health guidelines for the care and use of laboratory animals and with the recommendations of the Weatherall Report.

### Behavioral task

**Figure 1:**
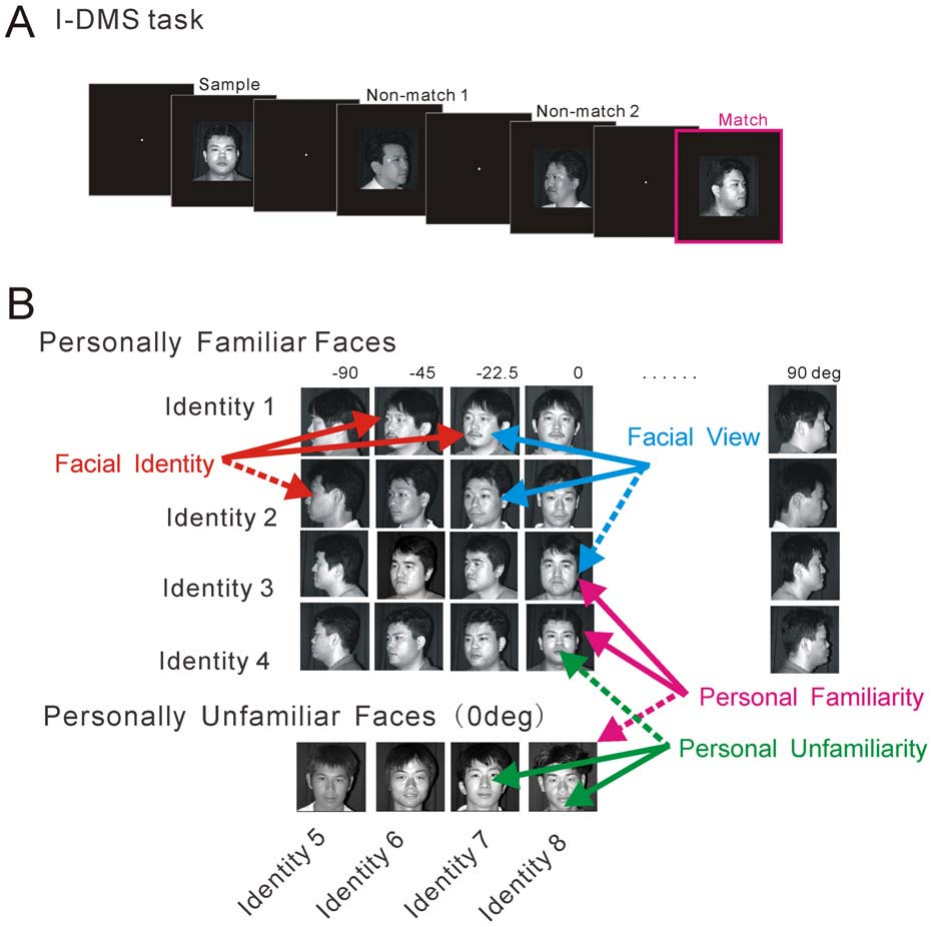
Behavioral task and Concept. **A.** Delayed matching-to-sample task based on identification (*I-DMS*) task, which was a version of the sequential delayed matching-to-sample task; a sample (480 ms) was presented after each monkey fixated a fixation point (*FP*, 0.2° diameter) that appeared at the center of the display. Then, test (match or non-match 480 ms) stimuli were presented after an inter-stimulus delay (992 ms). Intervening (non-match) stimuli were presented 0 to 3 times until a match finally appeared. Sample faces were always in the frontal view (0°), whereas a test face was one of 7 faces viewed from one of 7 different angles (from the *left to right* profile: −90, −45, −22.5, 0, 22.5, 45, and 90°). Both animals were required to identify the same person given in the sample; and if the test stimulus was a match, the monkey was trained to push a lever to obtain juice. Eye position was monitored using a scleral search coil during the *I-DMS* task, and the size of the eye control window was 2.0°. Visual stimuli were in 256 gray scale, 10–15×10–15° in size, and were presented at the center of the display with FP; thus all of the stimuli were within the receptive fields that were mapped before the experiments. **B.** Schematic description of the paradigm. Four types of comparison were made for the neuronal responses to the match stimuli. In the first comparison (magenta), the correlation coefficients between all possible personally familiar frontal face pairs (6 pairs) and the correlation coefficients between all possible personally familiar and unfamiliar frontal face pairs (16 pairs) were compared with zero. In the second comparison (green), the correlation coefficients between all possible personally unfamiliar frontal face pairs (6 pairs) and the correlation coefficients between all possible personally familiar and unfamiliar frontal face pairs (16 pairs) were compared with zero. In the third comparison (red), the correlation coefficients between all possible personally familiar face pairs of the same facial identity but in different facial views (84 pairs) and the correlation coefficients between all possible personally familiar face pairs of different facial identities in different facial views (252 pairs) were compared with zero. In the fourth comparison (cyan), the correlation coefficients between personally familiar face pairs in the same facial view but of different facial identities (42 pairs) and the correlation coefficients all possible personally familiar face pairs of between different facial views and of different facial identities (252 pairs) were compared with zero.

The monkeys were trained to perform a version of a sequential delayed matching-to-sample task requiring the identification of a face (I-DMS task **Fig. 1A**); this behavioral task was the same as that described in our preceding paper [16], [37]. In the I-DMS task, a sample (480 ms) stimulus was presented to the animal after fixation on a small point, and test (match or non-match, 480 ms) stimuli were subsequently presented to the subject after a period of inter-stimulus delay (992 ms). Eye position was monitored using the scleral search coil technique, and the size of the eye control window was ±2.0° [38]. Two types of facial stimuli were used in the experiment. The first type consisted of images of faces of people with whom the monkeys were already familiar; these people were laboratory staff involved in the daily care of the subjects; we call this type of face “personally familiar”. For the first type, the sample face was always presented in the frontal view (0°) but the match face was one of seven faces viewed from one of seven different angles (from *left* to *right* profile: −90, −45, −22.5, ±0, +22.5, +45, and +90°). The second type consisted of images of the faces of people with whom the monkeys were unfamiliar in their real life; we call this type of face “personally unfamiliar”. For the second type, the sample face was always presented in the frontal view (0°) and the match face was also presented in the frontal view (0°). We used only the frontal faces as personally unfamiliar matches because in the I-DMS task the monkeys could hardly learn to generalize the unfamiliar faces presented in different views as unique identities, as we discussed in our preceding paper [16]. We also used four abstract patterns as non-facial stimuli. The neutral abstract patterns were used as the control stimuli. Therefore, in the present experiment, any of 28 personally familiar faces (4 identities×7 views), 4 personally unfamiliar faces (other 4 identities×1 view), or 4 abstract patterns was used as the match in the I-DMS task. In half of the non-match presentations in each recording session, a non-match face or pattern was chosen from the 32 faces (28 personally familiar and 4 personally unfamiliar faces) or 4 abstract patterns which could be used as a match in the I-DMS task; in the other half of the non-match presentations, it was chosen from 112 faces or 16 patterns which were not used as the match. All visual stimuli were presented within the center of the receptive field of each recorded neuron, each of which had been mapped in advance of the experiment. In addition, the stimuli were typically centered on the fixation point, and the size of the images was 10–15×10–15°. The computer generating the visual stimuli was controlled by the standard laboratory real-time experimental system REX [39].

### Electrophysiological procedures

The procedures used for electrophysiological recording and data analysis have been described in detail in our preceding paper [16], [17]. In brief, these procedures were carried out as follows. During the experiment, a grid was placed within the recording cylinders [40] to facilitate the insertion of stainless steel guide tubes through the dura to a depth about 15–20 mm above the AIT. At the beginning of each recording session, the guide-tube stylet was removed and an epoxy-coated tungsten microelectrode (FHC, 1.0–1.5 MΩ at 1 kHz) was inserted. The electrode was advanced using a stepping microdrive, while neuronal activity was monitored to establish the relative depth of the landmarks, including the layers of gray and white matter, and to determine the properties of the neuronal responses. For all monkeys, we used 3D-MRI rendering to place an electrode into the AIT [41]. The positions of the AIT and of the recording sites were checked by MRI during the experiment, and these MR images included a marker (tungsten, 500 µm diam.); we verified the calculated recording sites with reference to the coordinate of the marker.

### Data analysis

In this study, we primarily analyzed single-neuron activity in response to match stimuli, i.e., during the period 64–496 ms after the onset of each (the lag time of 64 ms was based on the minimum response latency of neurons). Control firing was measured during the 208-ms period before the sample stimulus was presented. The time periods for the analyses were equal to the time periods as used in our previous report [16], [37]. Offline data analysis included spike density functions that were created by replacing the spikes with Gaussian pulses of a width corresponding to a 10-ms s.d. using the method of MacPherson and Aldridge [42], as implemented by Richmond et al. [43]. Neuronal responses to 36 match stimuli (28 personally familiar faces of 4 identities×7 views, 4 personally unfamiliar frontal (0°) faces and 4 abstract patterns) were used for the analysis. It should be noted that the activities of all the face-responsive neurons recorded in the AITv that were tested by both personally familiar and unfamiliar faces and satisfied the criteria (i.e., with significantly larger visual responses to faces than to abstract patterns), were used for the analysis; no selection beyond these criteria was made.

### Receiver's operating characteristics (ROC) analysis on individual neuron data

To investigate the stimulus selectivity of individual neurons, we analyzed the ROC curves based on the firings of each neuron in a given period. For the analysis of selectivity based on personal familiarity, the ROC curves were computed from RR*_familiar_* and RR*_unfamiliar_*, where RR*_familiar_* indicates the distribution of firings in response to all of the personally familiar faces in the frontal view, while RR*_unfamiliar_* indicates the distribution of firings in response to all of the personally unfamiliar faces presented in the frontal view. Here, we compared only the frontal view to remove the effects of facial views other than the frontal view. The area under the ROC curve (AUC) was then calculated and designated as AUC_personal familiarity/unfamiliarity_. For the analysis of selectivity upon facial identity of personally familiar faces, the ROC curves were computed from RR*_i_* and RR*_i_*, where RR*_i_* indicates the distribution of firings responding to facial identity *i* in all of the facial views while RR*_i_* indicates the distribution of firings responding to identities other than *i* in all of the facial views. Then the AUC was calculated for the four personally familiar identities and their maximum was designated as AUC_best identity, familiar faces_. Similarly, we also calculated the AUC_best view, familiar faces_ for the facial views of personally familiar faces that included all of the facial identities. On the other hand, for the analysis of selectivity during the facial identity of personally unfamiliar faces, the ROC curves were computed from RR*_ii_* and RR*_ii_*, where RR*_ii_* indicates the distribution of firings to personally unfamiliar facial identity *ii* in the frontal view, while RR*_ii_* indicates the distribution of firings in response to personally unfamiliar identities other than *ii* in the frontal view. Then the AUC was calculated for the four unfamiliar identities and their maximum was designated as AUC_best identity, unfamiliar faces_. The ROC curves based on the surrogate data, in which the relationship between visual stimuli and neural activities was shuffled, were also analyzed to estimate the significance of the original ROC curve.

### Correlation analysis for population-based data

To investigate the potential stimulus representations by the population of neurons, we performed a correlation analysis like that we executed in our previous studies [16], [17]. The neuronal responses were normalized to minimize the inherited influence of differences in the firing rate; for an individual neuron, the averaged neuronal response to each face was divided by the sum of all of the averaged neuronal responses. For all the combinations of two of the 28 familiar faces (_28_C_2_ = 378 pairs) and for all the combinations of two of the 8 frontal faces (_8_C_2_ = 28 pairs), the Pearson's correlation coefficient (*r*) between arrays of the normalized neuronal responses of all the face neurons in the population was calculated. The correlation coefficients were then transformed to Fisher's *z′* and the significance of differences between zero and the mean of *z*′-transformed *r* for pairs of a particular stimuli type, or the significance of differences between the means of *z′*-transformed *r* for pairs of particular stimuli types were analyzed using Student's *t*-test, at a significance level of *p* = 0.05.

Four types of comparison were made by the *t*-statistics as is schematically described in **Fig. 1B**. In the first comparison (magenta), the correlation coefficients between neuronal responses to 4 personally familiar frontal faces (_4_C_2_ = 6 pairs) and the correlation coefficients between neuronal responses to all possible personally familiar and unfamiliar frontal face pairs (_4_C_1_×_4_C_1_ = 16 pairs) were compared with zero (no correlation). The pairing of two magenta solid lines in **Fig. 1B** represents an example of a pairing between two personally familiar frontal faces, and the pairing of one magenta solid line and one magenta dashed line represents an example of a pairing between a personally familiar and an unfamiliar frontal face. In the second comparison (green), the correlation coefficients between neuronal responses to 4 personally unfamiliar frontal faces (_4_C_2_ = 6 pairs) and the correlation coefficients between neuronal responses to all possible personally familiar and unfamiliar frontal face pairs (_4_C_1_×_4_C_1_ = 16 pairs) were compared with zero (no correlation). The pairing of two green solid lines in **Fig. 1B** represents an example of a pairing between two personally unfamiliar frontal faces, and the pairing of one green solid line and one green dashed line represents an example of a pairing between a personally familiar and an unfamiliar frontal face. In the third comparison (red), the correlation coefficients between neuronal responses to personally familiar faces of the same facial identity but in different facial views (4*_7_C_2_ = 84 pairs) and the correlation coefficients between neuronal responses to personally familiar faces of different facial identities in different facial views (_28_C_2_-84-42 = 252 pairs) were compared with zero (no correlation). The pairing of two red solid lines in **Fig. 1B** represents an example of a pairing between two personally familiar faces of the same facial identity but in different facial views, and the pairing of one red solid line and one red dashed line represents an example of a pairing between two personally familiar faces of different facial identities in different facial views. In the fourth comparison (cyan), the correlation coefficients between neuronal responses to personally familiar faces in the same facial view but of different facial identities (7*_4_C_2_ = 42 pairs) and the correlation coefficients between neuronal responses to different facial views with different facial identities (252 pairs) were compared with zero (no correlation). The pairing of two cyan solid lines in **Fig. 1B** represents an example of a pairing between personally familiar faces in the same facial view but of different facial identities, and the pairing of one cyan solid line and one cyan dashed line represents an example of a pairing between two personally familiar faces of different facial identities in different facial views.

### Histological procedures

After the final recording session, several small marking lesions were created in the AIT by passing a 20- to 30-µA anodal current for 40 s through a tungsten microelectrode. Each animal was deeply anesthetized with an overdose of pentobarbital sodium (50 mg/kg, im) and perfused transcardially with heparinized 0.9% saline followed by 10% buffered formalin. The brains were removed and cut into 50-µm coronal sections through the target areas with a freezing microtome. Sections were stained with cresyl violet, and all sites marked by an electrical lesion were carefully verified microscopically. The location of each recording site was calculated by comparing the stereotaxic coordinates of the recording sites with those of the lesions. MR images obtained during the experiment were compared with those showing the marking electrodes to verify the calculated recording sites. The reconstruction of the recording sites based on histological investigation and MRI confirmed that all of the responses of the face neurons used for this analysis were recorded from the AITv in the range of 17–24 mm anterior to the interaural line; most of these face neurons were located around the anterior middle temporal sulcus (AMTS).

## Results

The monkeys performed the I-DMS task with the performance range of 85–98% correct. A total of 257 visually-responsive neurons were recorded from the AITv areas of the 2 monkeys (186 from monkey A and 71 from monkey B). In the present study, we focus on the particular data set of face-responsive neurons, exposed experimentally to 28 personally familiar face views, 4 geometric patterns and 4 personally unfamiliar faces. As the result, a total of 41 face-responsive neurons in the AITv were registered for the in-depth analysis (29 from monkey A and 12 from monkey B); the activities of all of these neurons increased significantly in response to a match face in comparison to a control firing (paired *t*-test, *p*<0.05) and also showed significantly larger responses to faces (familiar or unfamiliar) than to any of the 4 geometric patterns (Student's *t*-test with Welch's correction, two-tailed, *p*<0.05).

### Individual neuron data

The selectivity of the face-responsive neurons in the AITv based on personal familiarity or unfamiliarity was analyzed by applying the Student's *t*-test to the activities in response to the match for either a personally familiar or unfamiliar frontal face. Twenty-one neurons showed a significant difference (Student's *t*-test with Welch's correction, two-tailed, *p*<0.05). Also, the selectivity of the AITv face-responsive neurons upon the identity and/or the viewing of the personally familiar faces was analyzed by two-way ANOVA with repeated measures (factors: facial view, facial identity, two-tailed, *p*<0.05). Twenty-two and 10 neurons showed a significant main effect of facial identity and facial view, respectively, while 11 showed the significant interaction of facial view×facial identity.

**Figure 2:**
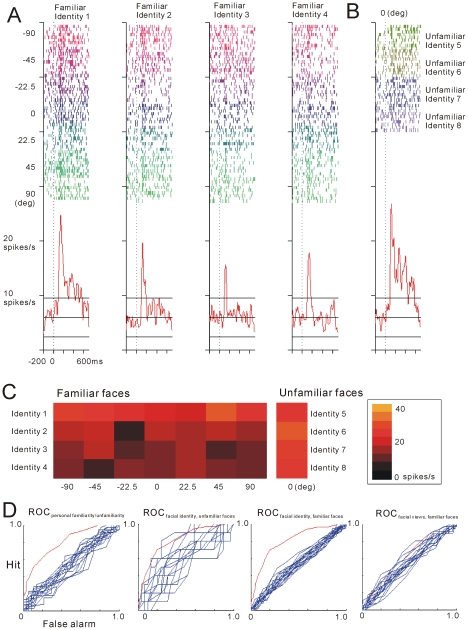
Example of an individual AITv neuron. **A.** Neuronal responses to a personally familiar face during the I-DMS task. Responses to 4 different identities are displayed in rasters, and spike density functions (s.d. = 10 ms) were aligned to the onset of the match (time = 0). Different raster colors indicate the 7 different facial views. Solid lines on the graphs indicate the mean firing rates during the control period (208-ms period before presentation of the sample faces) ± s.d. Different colors in the rasters indicate 7 different facial views. **B.** Neuronal responses to a personally unfamiliar face during the I-DMS task. Responses to 4 different identities are displayed in rasters and spike density functions with the same conventions as in **A.** Different colors in the rasters indicate 4 different facial identities. **C.** Neuronal responses to a personally familiar face of 7 facial views×4 facial identities (*left*) and those to a personally unfamiliar face of frontal view possessing of 4 facial identities (*right*), as summarized in the 2D color plot. **D.** ROC_personal familiarity/unfamiliarity_, ROC_best identity, unfamiliar faces_, ROC_best identity, familiar faces_, and ROC_best view, familiar faces_ curves (red) with 20 ROC surrogates (blue).

A representative example of AITv neurons is depicted in **Fig. 2A–D**. This neuron responded significantly more to personally unfamiliar faces than to personally familiar faces (Student's *t*-test, corrected, two-tailed, *p* = 0.1301×10^−5^). For the personally familiar faces, this neuron showed a significant main effect of facial identity (F_3, 231_ = 29.74 [*p* = 0.26876×10^−15^]; two-way ANOVA, factors: facial view and facial identity), and the activities of this face neuron were tuned to identity 1 [post hoc test (Fisher PLSD), *p*<0.05]. The main effect of facial view was not significant (F_6, 231_ = 1.5 [*p* = 0.1798]), while the interactions between facial view and facial identity were significant (F_18, 231_ = 1.98 [*p* = 0.0115]). For the personally unfamiliar faces, this neuron showed no significant main effect of facial identity (one-way ANOVA: F_3, 35_ = 0.7657 [*p* = 0.5246]). Selectivity of the match activities was also investigated by ROC analysis and the results are shown in **Fig. 2D**: the AUC value and *p*-value obtained by z-test with 20 surrogates in each ROC curve were 0.8069 (*p* = 0.1631-x 10^−139^, AUC_personal familiarity/unfamiliarity_), 0.6310 (*p* = 0.8683, AUC_best identity, unfamiliar faces_), 0.8039 (*p* = 0.9651×10^−72^, AUC_best identity, familiar faces_), and 0.6157 (*p* = 0.0013, AUC_best view, familiar faces_), respectively.

**Figure 3:**
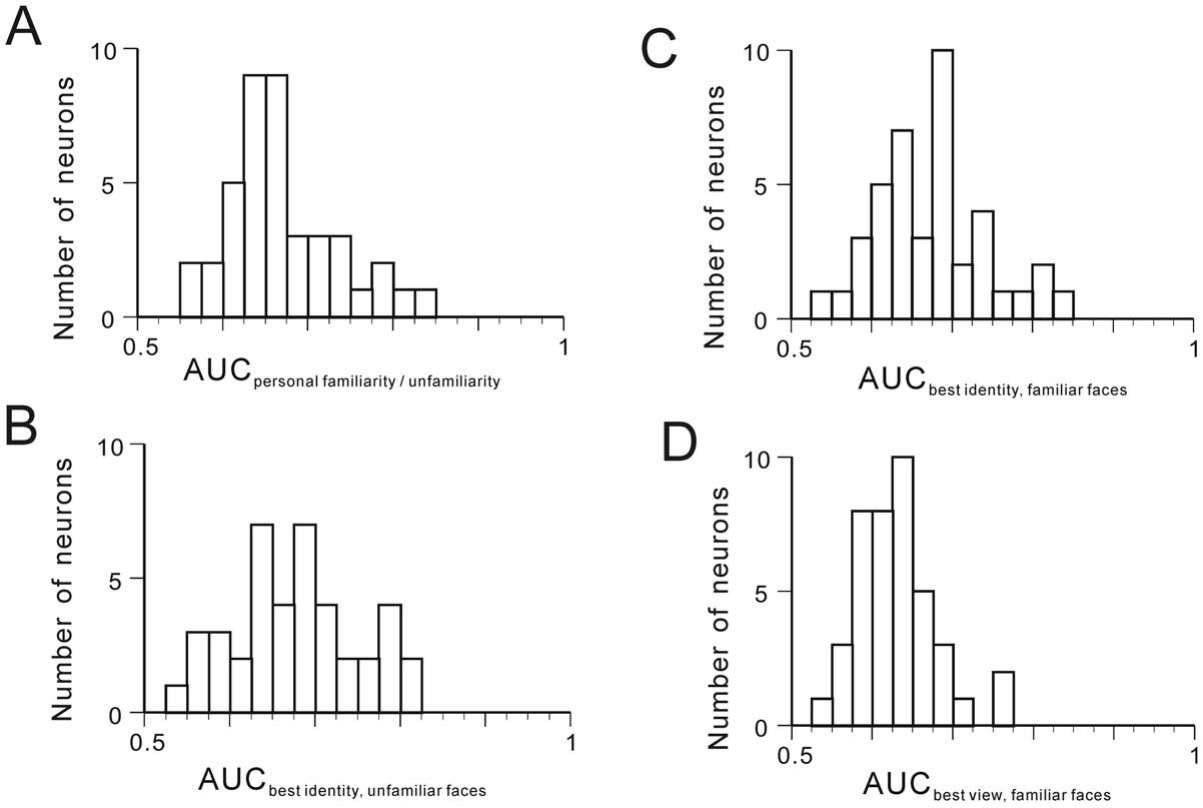
Individual selectivity to personal familiarity, facial identity and facial view: ROC analysis (N = 41). **A.** Frequency distribution of AUC_personal familiarity/unfamiliarity_. **B.** Frequency distribution of AUC_best identity, unfamiliar faces_. **C.** Frequency distribution of AUC_best identity, familiar faces_. **D.** Frequency distribution of AUC_best view, familiar faces_.

In **Fig. 3A** the distribution of the AUC_personal familiarity/unfamiliarity_ of the 41 neurons is shown. The mean±s.d. was 0.6728±0.0646. Thirty-nine (27 from monkey A and 12 from monkey B) of the 41 neurons showed significant difference from the 20 surrogate AUCs (*z*-test, *p*<0.05). In **Fig. 3B** the distribution of the AUC_best identity, unfamiliar faces_ of the 41 neurons is shown. The mean±s.d. was 0.6900±0.0909. In this analysis, 18 (13 from monkey A and 5 from monkey B) of the 41 neurons showed significant difference from the 20 surrogate AUCs (*z*-test, *p*<0.05). This implies that for personally unfamiliar faces, more than half of the samples did not show significant selectivity to facial identities. In **Fig. 3C**, the distribution of AUC_best identity, familiar faces_ of the sample in the present study is shown. The mean±s.d. was 0.6793±0.0740. In this analysis, 39 (28 from monkey A and 11 from monkey B) of the 41 neurons showed significant difference from the 20 surrogate AUCs (*z*-test, *p*<0.05). This implies that for personally familiar faces, most of the samples did show significant selectivity to facial identities. In **Fig. 3D**, the distribution of AUC_best view_ of the sample in the present study is shown. The mean±s.d. was 0.6313±0.0495. In this case, 32 (23 from monkey A and 9 from monkey B) of 41 showed significant difference from the 20 surrogate AUCs (*z*-test, *p*<0.05). The mean AUC_best identity, familiar faces_ was significantly larger than that of AUC_best view, familiar faces_ (Student's *t*-test, corrected, two-tailed, *p* = 0.00079), implying sharper selectivity of the samples to facial identities than facial views, for personally familiar faces.

### Population data

**Figure 4:**
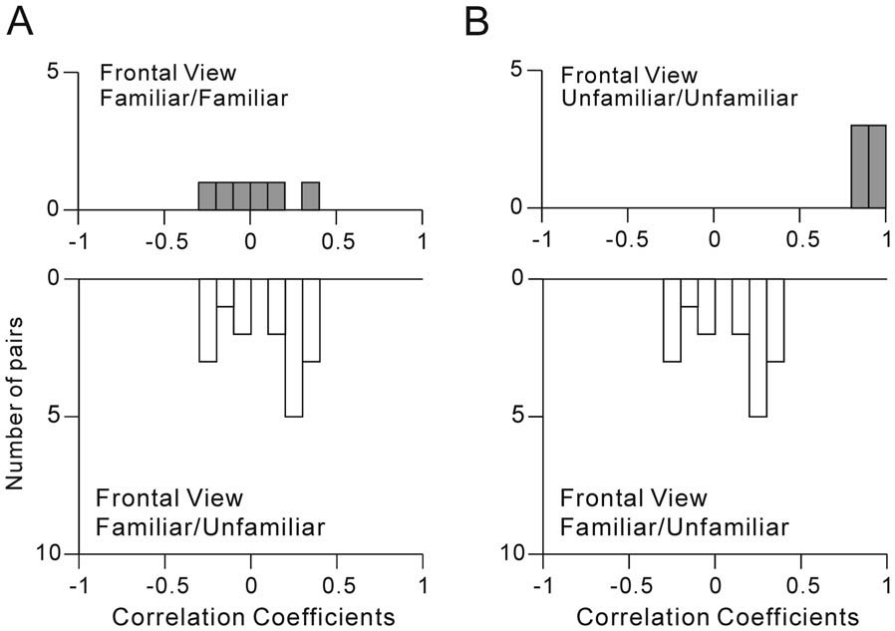
Neuronal population-based similarity measures for personal familiarity and unfamiliarity. **A.** Frequency distribution of the correlation coefficients between the neuronal responses to 2 personally familiar faces (6 pairs: *upper*) and frequency distribution of the correlation coefficients between the neuronal responses to a personally familiar and a personally unfamiliar face (16 pairs: *lower*). Only frontal faces were analyzed. **B.** Frequency distribution of the correlation coefficients between the neuronal responses to 2 personally unfamiliar faces (6 pairs: *upper*) and frequency distribution of the correlation coefficients between the neuronal responses to a personally familiar and a personally unfamiliar faces (16 pairs: *lower*). Only frontal faces were analyzed.

We calculated the correlation coefficients between responses of the 41 neurons (29 from monkey A and 11 from monkey B) to a pair of faces used as the match in the I-DMS task to analyze the similarity/dissimilarity relationship between the faces represented by the activities of a population of the face-responsive neurons. Results of the first comparison (**Fig. 1B**) are summarized in **Fig. 4A**. The frequency distribution of the correlation coefficients between possible pairs of the personally familiar, frontal faces possessing different identities (n = 6) is depicted in **Fig. 4A** **(upper)**. The mean±s.d. was −0.0116±0.2177, and was not significantly different from zero (Student's *t*-test, two-tailed, *z′*-transformed, *p* = 0.9148). The results indicate that the population of face neurons cannot represent personally familiar faces in the same frontal view as similar ones, with a probability significantly greater than chance. The frequency distribution of the correlation coefficients between personally familiar and unfamiliar frontal faces possessing different identities (n = 16) is depicted in **Fig. 4A** **(lower)**. The mean±s.d. was 0.0552±0.2114 and was not significantly different from zero (Student's *t*-test, two-tailed, *z′*-transformed, *p* = 0.3174). There was no significant difference between the means of the two distributions (Student's *t*-test, corrected, two-tailed, *z′*-transformed, *p* = 0.5494). The results indicate that the population of face neurons cannot represent a personally unfamiliar face and a familiar faces, both in the same frontal view, as similar to one another with a probability significantly greater than chance. In other words, the results indicated that personally unfamiliar frontal faces are differentiated from personally familiar frontal faces.

Results of the second comparison (**Fig. 1B**) are summarized in **Fig. 4B**. The frequency distribution of the correlation coefficients between personally unfamiliar, frontal faces possessing different identities (n = 6) is depicted in **Fig. 4B** **(upper)**. The mean±s.d. was 0.8417±0.0249 and was significantly different from zero (Student's *t*-test, two-tailed, *z′*-transformed, *p* = 0.3100×10^−4^). **Figure 4B** **(lower)** and **Fig. 4A** **(lower)** are identical graphs. There was a significant difference between the means of the two distributions, **Fig. 4B** **(upper)** and **Fig. 4B** **(lower)** (Student's *t*-test, corrected, two-tailed, *z′*-transformed, *p* = 0.1028×10^−5^). The results indicate that the population of face neurons can represent personally unfamiliar faces as similar to one another, with a probability significantly greater than chance. In **Fig. 4**, only the frontal faces were compared in the analysis, since only the frontal faces were used for the personally unfamiliar faces, as described in Methods (**Fig. 1B**).

**Figure 5:**
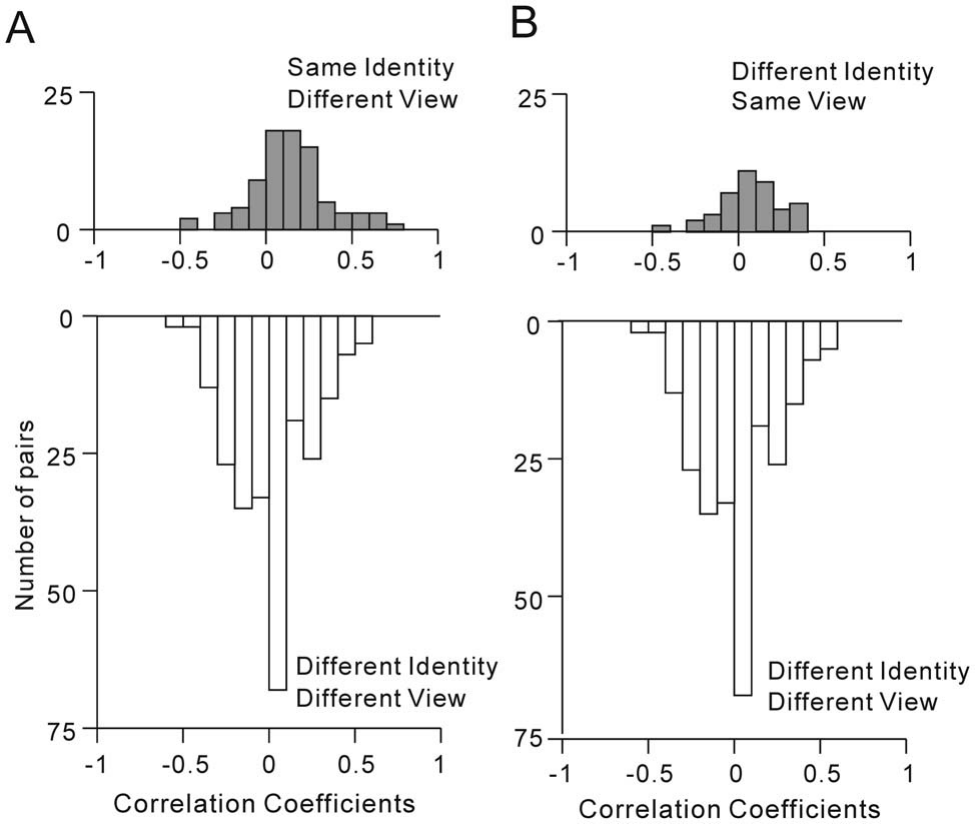
Neuronal population-based similarity measures for facial identity and facial view of personally familiar faces. **A.** Frequency distribution of the correlation coefficients between the neuronal responses to 2 personally familiar faces possessing the same identity but presented in different views (84 pairs: *upper*) and frequency distribution of the correlation coefficients between 2 personally familiar faces possessing different identities and presented in different views (252 pairs: *lower*). **B.** Frequency distribution of the correlation coefficients between the neuronal responses to 2 personally familiar faces possessing different identities but presented in the same view (42 pairs: *upper*) and frequency distribution of the correlation coefficients between 2 personally familiar faces possessing different identities and presented in different views (252 pairs: *lower*).

Then, we further analyzed the representations of the personally familiar faces. Results of the third comparison (**Fig. 1B**) are summarized in **Fig. 5A**. In **Fig. 5A** **(upper)**, the frequency distribution of the correlation coefficients between personally familiar faces possessing the same facial identity but in different views (n = 84) is depicted. The mean±s.d. was 0.0918±0.2277 and was significantly different from zero (Student's *t*-test, two-tailed, *z′*-transformed, *p* = 0.5132×10^−3^). In **Fig. 5A** **(lower)**, the frequency distribution of the correlation coefficients between personally familiar faces possessing different facial identities and in different views (n = 252) is depicted. The mean±s.d. of the correlation coefficients was −0.0365±0.2155. The mean was negative and significantly different from zero (Student's *t*-test, two-tailed, *z′*-transformed, *p* = 0.0084). There was a significant difference between the means of the two distributions (Student's *t*-test, corrected, two-tailed, *z′*-transformed, *p* = 0.1918×10^−4^). The results indicate that the population of AITv face neurons can represent personally familiar faces possessing the same facial identities but presented in different facial views as similar to one another, with a probability significantly greater than chance. In other words, the population of AITv face neurons can represent the identities of personally familiar faces in a manner independent of the view.

The results of the fourth comparison (**Fig. 1B**) are summarized in **Fig. 5B**. In **Fig. 5B** **(upper)**, the frequency distribution of the correlation coefficients between personally familiar faces in the same view but possessing different identities (n = 42) is depicted. The mean±s.d. was 0.0232±0.1758 and was not significantly different from zero (Student's *t*-test, two-tailed, *z′*-transformed, *p* = 0.4123). **Figure 5A** **(lower)** and **Fig. 5B** **(lower)** are identical graphs. There was no significant difference between the means of the two distributions (Student's *t*-test, corrected, two-tailed, *z′*-transformed, *p* = 0.0565). The results indicate that the population of AITv face neurons cannot represent personally familiar faces in the same view but possessing different facial identity as similar to one another, with a probability greater than chance. In other words, the population of AITv face neurons cannot represent the views of personally familiar faces in a manner independent of identities.

## Discussion

Personal familiarity of faces is a critical constraint upon the face processing of primates. It has been shown by a number of behavioral studies that behavioral measures related to the face recognition of personally familiar and unfamiliar faces are quite different [18], [19]. The possibility of different neural mechanisms for the processing of personally familiar and unfamiliar faces has been suggested by EEG [25], [27], MEG [26], PET [20], [22] and functional MRI studies [21], [23], [24], [44] in humans. The results in the present study, using a single-cell recording technique in monkeys, outlined neural representations for the personally familiar and unfamiliar faces in the AITv area, which is considered to be the area crucial for face identification in monkeys [16], [17], [37].

Single-cell recording studies in monkeys have so far delineated a substantial impact of repetitive visual learning on neural representations of visual items in the AIT [28]–[36], [45]–[48]. In accord with the substantial neural changes that have been reported, the results in the present study revealed that, in the pattern of activities of the population of AITv neurons, the personally unfamiliar faces were differentially represented from personally familiar faces, and more importantly, similarly across their facial identities (**Fig. 4**). Whereas for personally familiar faces, our results indicated that, different identities were differentially represented irrespective of their views while different views were not represented independently of their facial identities by the same populations of AITv neurons (**Fig. 5**), consistent with our previous report.

There remains a possibility that differences in cognitive demands between personally familiar and unfamiliar face stimuli affected the present results, since the behavioral task used in the present study required some generalization of a unique facial identity across the facial views in the case of personally familiar faces but not at all in the case of personally unfamiliar faces. Because it was quite difficult to get the monkeys to achieve generalization of unique facial identities across facial views using personally unfamiliar faces and perform the I-DMS task reliably, as we pointed out in the Methods section and also in our previous report [16], we did not use facial views other than the frontal view for the personally unfamiliar faces in the present study.

It should be noted that the term “familiarity” may imply different subsets of phenomena. Personal familiarity in this case applies when a subject knows the person being viewed in daily life, and has personal interaction with that person. This applies, for instance, to teachers for children, to animal caretakers for monkeys, etc. This kind of personal relationship is usually accompanied with autonomic or emotional responses, which is distinctive from the other subsets of familiarity [25], [49], since it potentially activates the limbic brain structures. Another case of familiarity is that the subject knows the person because that person is famous; in such a case, there are no needs for personal interaction between the subject and the person. We call this “public familiarity”. Public familiarity applies for, say, well-known television personalities. Yet another case of familiarity is when a subject knows the person only by repeated exposure to his or her face as a visual stimulus. This case is designated as visual familiarity [50]. Visual familiarity applies, for instance, to photographs of unfamiliar persons to which the subject has been repeatedly exposed. In this framework, we should like to emphasize that the 28 *familiar* faces that were used in the present study were of individuals personally familiar to the animals. We also should like to emphasize that the 4 *unfamiliar* faces in the present study were personally unfamiliar but visually familiar. In many of the psychological studies on the familiarity of faces that have been published to date, public familiarity is usually used to define the familiarity of faces. However, because we considered that it would be somewhat nonsensical to measure the public familiarity for monkeys, we focused on the difference between two extremes in familiarity, i.e., personal familiarity and visual familiarity. Our results indicated that the population of AITv face neurons does not distinguish among different facial identities of *personally unfamiliar* (but *visually familiar*) faces with the same view when that view is frontal. On the other hand, the population does differentiate among facial identities of *personally familiar* faces with the same view when that view is a −90 to 90 degree view.

At this point, two possible interpretations of the differentiation of personally unfamiliar faces remain. One is that the personally unfamiliar but visually familiar faces are represented as a single category that is distinct from the personally familiar facial identities, and this category can also be differentiated from the visually unfamiliar faces. Another possibility is that the personally unfamiliar but visually familiar faces are not represented as a distinct category that can be differentiated from the faces that are only visually unfamiliar. In other words, the visually unfamiliar faces might behave in a manner similar to the personally unfamiliar but visually familiar faces. Specifying whether or not the animals could form a distinct category for the personally unfamiliar but visually familiar faces is important for understanding the relationship between the visual expertise and the organization of neural representations by the population of face-responsive neurons in the AITv. It has been reported that an increase in visual expertise is able to cause substantial changes in the neural activation of the face-related area in humans [9]. However, at this stage we cannot confirm this point, since we did not use trial-unique, visually unfamiliar faces in the stimuli battery in the present study; further studies are required.

Moreover, we would like to note that this study has several limitations due to the practical difficulties of using non-human primates as animal subjects in this kind of experiment. Although this is not unusual for monkey single-cell recording studies, the study is based on a small sample size (N = 2) and was conducted only on female subjects. Nonetheless, we would emphasize that the results obtained for monkey A and monkey B were quite consistent in the present study. In particular, the results for AUC_personal familiarity/unfamiliarity_ and AUC_best identity, unfamiliar faces_ obtained by ROC analysis were quite similar between monkey A and monkey B (*see* the texts for **Fig. 3**). Also, in the analysis of the representations by the population of face-responsive neurons, a substantial number of neurons from each of the monkeys contributed (*see* the texts for **Fig. 4** and **5**). Therefore, we think that the results are generalizable. With regard to potential gender differences, especially those phenomena associated with affective bonding, we should be very careful to note the possibility that the specificity in gender might have affected the generalizability of our results.

Some researchers have suggested that disturbance in the recognition of personal familiarity or unfamiliarity underlies delusional misidentification syndromes such as the Capgras delusion [51]–[53]. The Capgras delusion is a delusion that a very familiar person, such as close friend, spouse, parent, or other close family member has been replaced by an impostor with identical looks. Ellis and Young [51] hypothesized that the patients with Capgras delusion may have a mirror image of another very characteristic syndrome, prosopagnosia, which indicates in its narrow sense a cognitive inability to identify familiar individuals by faces. Ellis and Young [51] suggested that, while their conscious ability to recognize faces was intact, patients with Capgras delusion might have some damage to the system that produces the automatic emotional arousal to familiar faces, and this creates the bizarre experience of recognizing someone while feeling that something is not quite right about them.

Previously, our findings suggested that the population of face-responsive neurons in the AITv area representing facial identities might be closely related to the underlying mechanisms of prosopagnosia [16], [17]. Similar results were obtained from other laboratories [15]. On the other hand, the findings in the present study disclosed another important aspect of neuronal representations by the population of face-responsive neurons in the AITv area. The results regarding the neural representations of personal familiarity or unfamiliarity seem to identify a component in the neural system that comprises some essential aspect of normal face recognition and therefore may underlie the Capgras delusion.
